# Nervous System

**Published:** 1871-02

**Authors:** G. F. Patton

**Affiliations:** Cincinnati, O.


DENTAL REGISTER.

VOL. XXV.] FEBRUARY, 1871. [NO. 2.

### Communications.

### NERVOUS SYSTEM.

Essay by G. F. Patton, Jan. 21st, 1870, Cincinnati, O. Read in Quiz Class. Dental College.

It is clear that whatever lives and moves must be supplied with muscular force, and that this force must be directed by some agency, causing it to act with regularity and in such a way as to benefit the machine of which it forms a part. Now as far as our investigations have enabled us to discover, every motion of living beings is directed by the same kind of apparatus, viz.: muscles, whose action is entirely regulated by nerves. Therefore we are justified in the hypothesis that all vital nature is supplied with appropriate nerves. Even vegetable life may be said to have its peculiar nervous arrangement, for we find the roots of plants killed by an agent acting on the leaves and branches. In some species of plants there is such an exquisite sensitiveness that a touch of a single leaf causes the entire foliage to shrink and withdraw. Therefore starting with the hypothesis that every vital organization has its proper nerves, we will examine that order belonging to the vertebrata, and particularly of man.

The general principles of nervous endowment are the same in all animals, even in its most primitive and simple form.

This system is seen to consist of a central organ, or a number of organs joined by commissures, and from this center nervous cords radiating. In all the vertebrated species the center is essentially this: a medullary cord. In man this is called the cerebro-spinal axis, from which are given off three distinct classifications of nerves. Those in the cranium; Those given off from the spinal cord; and those arising from the ganglionic or great sympathetic system, which extends along the outside of the spinal column. I propose to discuss particularly the action of the nerves, but it seems that without a preface of anatomy, the harmony of this beautiful structure can not be properly demonstrated.

The brain and spinal cord are composed of two kinds of substance, white and gray. In the brain the gray substance lies externally to the white, much as the bark covers the inner portion of a tree, hence it is called cortical. It is also called cineritious, from having the color of ashes. In the spinal cord, however, the gray substance forms the interior portion. Each of these (white and gray) portions has its peculiar function. At the base of the brain and about the fourth ventricle, from which the greater part of the cranial nerves arise, it will be seen that those having a sensitive function spring from the gray matter in that region. In the spinal cord, which we have found to consist of both gray and white matter, the sensitive roots of the spinal nerves arise in a tract of gray (Port. Lat. Sulcus) in connection with the posterior column. So we may safely say that the gray substance of the spinal cord and brain is the seat of sensation, or at least that it receives the sensitive impulse. The white portion is then left as the seat of motor action; and this conclusion is carried out in the cord where the white matter preponderates, and as the motor spinal nerves supply a much greater demand than the sensitive. As the motor nerves arise from the anterior lateral column, we have good grounds for supposing that the white substance presides over motion. We all understand that these conclusions must be based on experiments and hypothesis drawn from these experiments. So while there is a chance that these conclusions may be wrong, there is an almost certainty that they are correct. The manner in which the nerves act is peculiar, and they owe their power of transmitting impulses to some inherent principle that we can have little hope to explain. The nerves may be compared to a certain class of philanthropists; they work entirely for the good of the general system and appropriate no benefit to themselves, but they are only willing to work in their own legitimate capacity. If one artery fails, another will supply the deficiency thus created. If one of the organs of digestion is paralyzed, another will try to assume its function. *Not so* the nerves. Although in the spinal nerves the sensor and motor filaments lie mingled in the same sheath and are, perhaps, distributed to the same parts, yet if the motor root be divided, the part which it supplies is paralyzed so far as motion is concerned, but the sensitive portion of the nerve continues to carry impressions to the brain just as if its motor brother was not injured, thus showing a perfect disregard of all except its own *duty*.

The provision of nature is, that the sensitive nerves shall only transmit impulse or impressions to the brain, and the motor convey the dictates or commands of the will to the muscles. For example, should the little finger come in contact with a red hot body, the superficial Palmer branch of the Ulnar nerve immediately telegraphs to the brain that the part over which it is standing guard is being imposed upon; the brain immediately telegraphs back by a motor nerve to the muscles of that region to remove the finger from contact with the offending object. This is done forthwith, but the finger must wait until all this communication takes place before its suffering can be relieved. This is all done, however, with electric rapidity.

The cerebro-spinal center and particularly the brain, is like the capital city of a great government. There are its various chiefs of departments in the organs of special sense, the sympathetic ganglia, the cranial nerves, and the plexuses of the spinal nerves; each of these departments has its peculiar affairs, which are under the supervision of the smaller branches coming from the ganglion, plexis, &c. The olfactory nerve has its detective officers distributed to the Schinderian membrane to watch that no odors or effluvia which are offensive or injurious can have admission. The optic nerve stands with its retinal expansion of sentinels, to watch in the light houses, and the other two of special sense act similarly in their respective functions. The pneumogastric is a special officer, who must look to the performance of the heart, lungs, and stomach; it also must have an eye on the spleen and pancreas, gall cyst and liver.

The spinal nerves are mail routes and telegraph lines to more distant parts of the country. In each department there is so much business to be transacted that there must be a separate mail route or wire for the intelligence passing to the capital, and the instruction from the capital to the organs in the different parts. Here we have the sensitive nerves bringing the news to the brain and the motor nerves taking instructions from the brain to the muscles or other organs. The communication from the sensor to the motor nerves can be excited, even after death, by electric shocks, by corrosive chemical agents, and by mechanical irritation—the precise manner in which the motor nerves affect the muscles, can never be known. The muscles themselves are possessed of some principles that cause them to respond to the action of galvanism and the other exciting agents above mentioned. But what the nature of these influences is, yet remains a subject of conjecture. The great sympathetic system of nerves, is one that is independent both of the cranial and spinal, though communicating with both. It consists of a series of ganglions extending along the outside of the spinal column, from the head to the coccyx. Its branches go to the internal organs and viscera. It presides over the action of those organs which are not under the control of the will, such as the heart in its diastole and systole and the secreting organs of the whole body. Its service to the great government is performed in mystery and secrecy. It has entire control over its districts, and no simple order from the imperious will can suspend its action. It is one of the old style of workmen who performed their work promptly and perfectly; but it can not be made subservient to the same power that rules the other senitors of the brain. It does all that is required of it, but must not be questioned, nor commanded. It dilates or contracts the iris, so as to graduate the intensity of light admitted, to the most minute shade. It regulates the rise and fall of the chest, controls the beat of heart, the secretions of all the glands. It knows by its communicators the events transpiring around and regulates its action accordingly.

